# Systemic bioinformatics analysis of recurrent aphthous stomatitis gene expression profiles

**DOI:** 10.18632/oncotarget.22347

**Published:** 2017-11-10

**Authors:** Jian Wu, Zheng-Ping Chen, An-Quan Shang, Wei-Wei Wang, Zong-Ning Chen, Yun-Juan Tao, Yue Zhou, Wan-Xiang Wang

**Affiliations:** ^1^ Department of Laboratory Medicine, The First People’s Hospital of Yancheng City, Yancheng 224006, Jiangsu, China; ^2^ Clinical Medicine School, Jiangsu Vocational College Medicine, Yancheng 224002, Jiangsu, China; ^3^ Department of Laboratory Medicine, Tongji hospital of Tongji University, Shanghai 200092, Shanghai, China; ^4^ Department of Pathology,The Sixth People’s Hospital of Yancheng City, Yancheng 224005, Jiangsu, China; ^5^ Department of Laboratory Medicine, Yancheng TCM Hospital Affiliated To Nanjing University of Chinese Medicine, Yancheng 224001, Jiangsu, China

**Keywords:** visualization and integrated discovery (DAVID), gene expression omnibus (GEO), limma, immune, RAS

## Abstract

Recurrent aphthous stomatitis (RAS) represents the most common chronic oral diseases with the prevalence ranges from 5% to 25% for different populations. Its pathogenesis remains poorly understood, which limits the development of effective drugs and treatment methods. In this study, we conducted systemic bioinformatics analysis of gene expression profiles from the Gene Expression Omnibus (GEO) to identify potential drug targets for RAS. We firstly downloaded the gene microarray datasets with the accession number of GSE37265 from GEO and performed robust multi-array (RMA) normalization with affy R programming package. Secondly, differential expression genes (DEGs) in RAS samples compared with control samples were identified based on limma package. Enriched gene ontology (GO) terms and Kyoto Encyclopedia of Genes and Genomes (KEGG) pathways of DEGs were obtained through the Database for Annotation, Visualization and Integrated Discovery (DAVID). Finally, protein-protein interaction (PPI) network was constructed based on the combination of HPRD and BioGrid databases. What’s more, we identified modules of PPI network through MCODE plugin of Cytoscape for the purpose of screening of valuable targets. As a result, 915 genes were found to be significantly differential expression in RAS samples and biological processes related to immune and inflammatory response were significantly enriched in those genes. Network and module analysis identified FBXO6, ITGA4, VCAM1 and etc as valuable therapeutic targets for RAS. Finally, FBXO6, ITGA4, and VCAM1 were further confirmed by real time RT-PCR and western blot. This study should be helpful for the research and treatment of RAS.

## INTRODUCTION

Despite the rapid development of anticatarrhals and treatment methods, recurrent aphthous stomatitis (RAS) still represents the most common chronic oral disease, which suffered from a long-term painful and characterized by a yellowish ulcer and with an erythematous halo that heals spontaneously [1–3]. The prevalence of RAS could variy from 5% to 25% for people with different diet habits and come to the highest for people between 10-40 years of age [4]. Several inducing factors, such as work-rest schedule, stress, smoking, stress, and etc are summaried, and these factors may joint to influence the pathological process. In addition, previous study also showed that RAS could be induced by the abnormal mucosal cytokine cascade, which precipitated by clinical or subclinical trauma and leading to an enhanced cell-mediated immune response directed toward focal areas of the oral mucosa [5, 6]. What’s more, Wray et al [7] even reported that RAS could induce the initiation of oral squamous cell carcinoma for susceptible population. However, the underlying etiology and pathogenesis remain poorly understood. The progress of molecular has accelerate the emergence of novel drugs and therapeutic methods for the pain relase and reduction of RAS recurrence frequency, while, there is still no effective methods for its healing.

In the past decades, gene microarray has been as a valid method for the parallel quantification of expression values for hundreds, and even thousands of genes at once time [8]. Besides, the application of gene microarray has also promoted the identification of multi disease-related genes, including RAS related genes [9]. In this study, we conducted systemic bioinformatics analysis, including screening of differential expression genes (DEGs), functional enrichment analysis, and network analysis, of gene expression profiles of RAS and adjacent normal tissues which detected through gene microarray for the identification of potential RAS related genes. Besides, several potential RAS related genes identified by bioinformatics were further confirmed by real time RT-PCR. Our study should shed light on the mechanisms of RAS and provide valuable biomarkers for RAS.

## RESULTS

### Different expression genes (DEGs)

**Figure 1:**
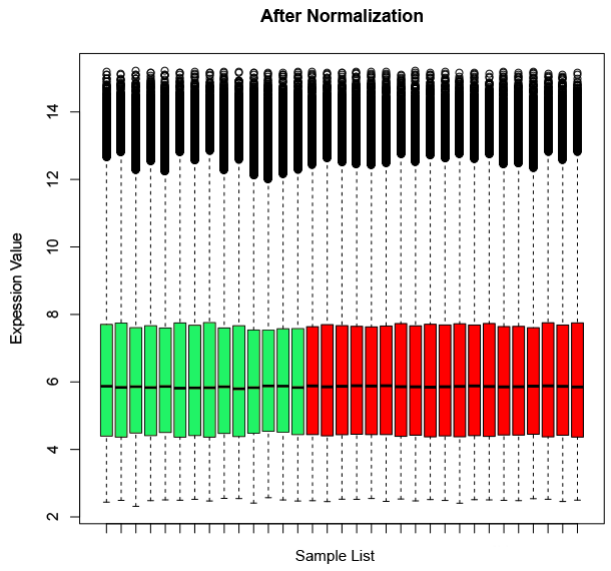
Overall mRNA level of all probesets in the microarray after normalized through affy package The normalized expression values are comparable among all of the samples which should be suitable for the following analysis.

Figure 1 illustrated the relative mRNA levels in all of the samples after normalization. Based on the criteria of FDR < 0.05 and fold change > 2, we identified a total of 915 DEGs in RAS samples compared with adjacent non-ulcer tissue. Strikingly, more than three quarters (707/915) of DEGs were found to be up-regulated in RAS, while, only 208 genes were down-regulated.

### Enriched functions

**Figure 2:**
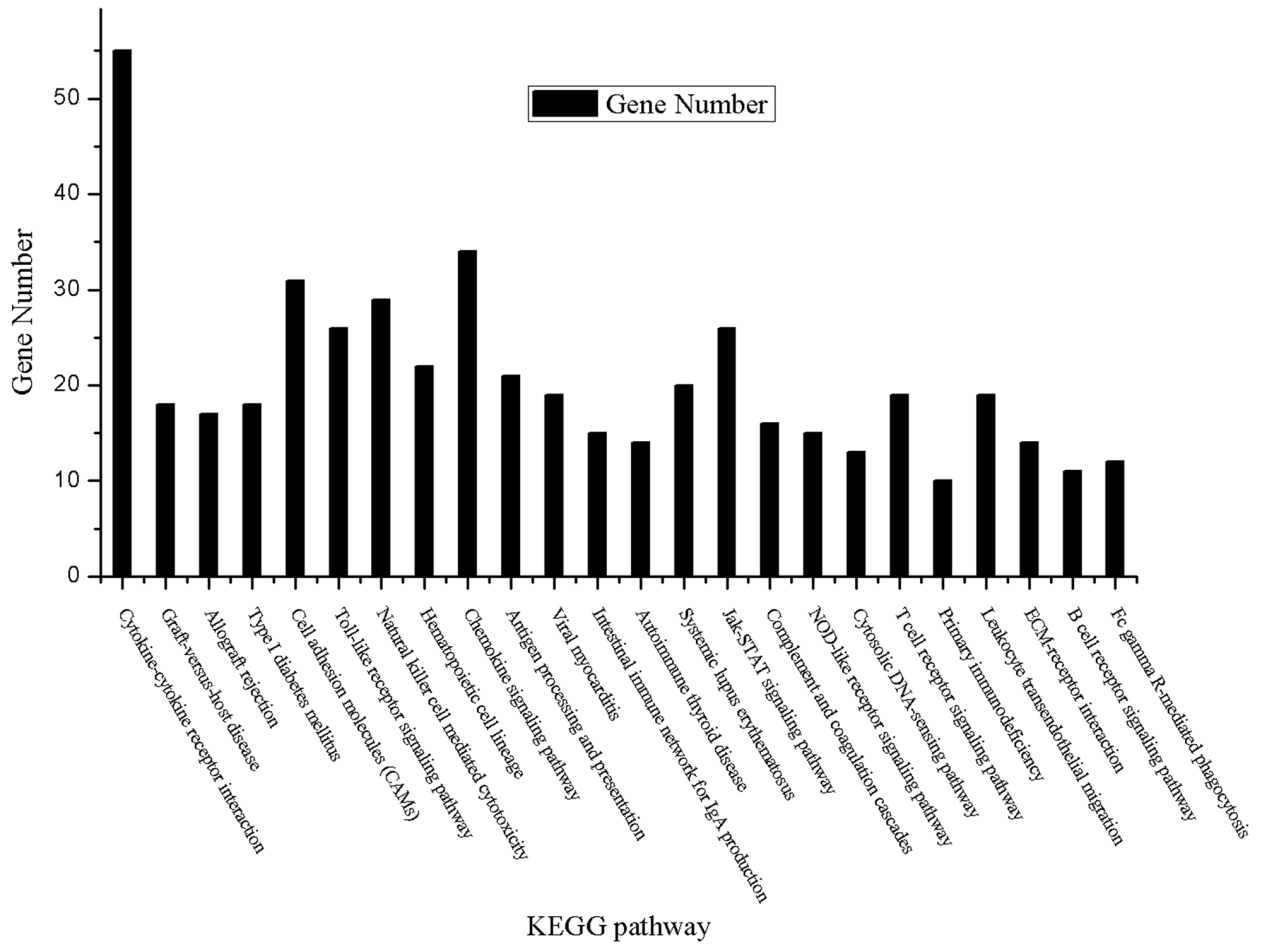
Significantly enriched KEGG pathways and corresponding DEG number contained in them

A total of 215 GO terms and 24 KEGG pathways were found to be significantly enriched in the DEGs. Table 1 shows the top 20 most significantly enriched GO terms which mainly involved in immune and inflammatory response. The full list of KEGG pathways were shown in Table 2. Consistent with the results of GO terms, most of KEGG pathways are closely associated with immune and inflammatory response, such as Cytokine-cytokine receptor interaction, Natural killer cell mediated cytotoxicity. Figure 2 illustrated the significantly enriched KEGG pathways and their hits number, i.e. DEGs contained in them.

**Table 1:** The top 20 GO terms of DEGs according to P value

Category	GOID	GO Name	Gene Number	Pvalue
BP	GO:0006955	immune response	180	4.25E-84
BP	GO:0006952	defense response	137	1.41E-53
BP	GO:0009611	response to wounding	105	1.09E-35
BP	GO:0006954	inflammatory response	82	1.50E-35
CC	GO:0044421	extracellular region part	138	8.00E-29
CC	GO:0005576	extracellular region	214	6.49E-27
CC	GO:0005615	extracellular space	109	3.63E-26
BP	GO:0002684	positive regulation of immune system process	60	6.30E-26
BP	GO:0006935	chemotaxis	44	1.18E-20
BP	GO:0042330	taxis	44	1.18E-20
BP	GO:0050778	positive regulation of immune response	42	1.20E-20
BP	GO:0001775	cell activation	58	4.74E-20
BP	GO:0048584	positive regulation of response to stimulus	52	1.02E-19
BP	GO:0045321	leukocyte activation	52	3.21E-19
BP	GO:0009615	response to virus	34	7.35E-18
BP	GO:0042110	T cell activation	33	7.11E-15
BP	GO:0050867	positive regulation of cell activation	31	8.07E-15
BP	GO:0046649	lymphocyte activation	41	1.59E-14
BP	GO:0045087	innate immune response	34	1.78E-14
MF	GO:0005125	cytokine activity	40	2.47E-14

**Table 2:** The KEGG pathways of DEGs

Category	Pathway Name	Gene Number	Pvalue
KEGG_PATHWAY	Cytokine-cytokine receptor interaction	55	1.07E-14
KEGG_PATHWAY	Graft-versus-host disease	18	8.65E-11
KEGG_PATHWAY	Allograft rejection	17	2.21E-10
KEGG_PATHWAY	Type I diabetes mellitus	18	3.59E-10
KEGG_PATHWAY	Cell adhesion molecules (CAMs)	31	1.18E-09
KEGG_PATHWAY	Toll-like receptor signaling pathway	26	4.72E-09
KEGG_PATHWAY	Natural killer cell mediated cytotoxicity	29	2.65E-08
KEGG_PATHWAY	Hematopoietic cell lineage	22	1.09E-07
KEGG_PATHWAY	Chemokine signaling pathway	34	1.38E-07
KEGG_PATHWAY	Antigen processing and presentation	21	2.78E-07
KEGG_PATHWAY	Viral myocarditis	19	4.93E-07
KEGG_PATHWAY	Intestinal immune network for IgA production	15	2.01E-06
KEGG_PATHWAY	Autoimmune thyroid disease	14	1.85E-05
KEGG_PATHWAY	Systemic lupus erythematosus	20	2.03E-05
KEGG_PATHWAY	Jak-STAT signaling pathway	26	2.49E-05
KEGG_PATHWAY	Complement and coagulation cascades	16	3.35E-05
KEGG_PATHWAY	NOD-like receptor signaling pathway	15	3.91E-05
KEGG_PATHWAY	Cytosolic DNA-sensing pathway	13	1.98E-04
KEGG_PATHWAY	T cell receptor signaling pathway	19	2.27E-04
KEGG_PATHWAY	Primary immunodeficiency	10	3.42E-04
KEGG_PATHWAY	Leukocyte transendothelial migration	19	6.92E-04
KEGG_PATHWAY	ECM-receptor interaction	14	0.003302
KEGG_PATHWAY	B cell receptor signaling pathway	11	0.025533
KEGG_PATHWAY	Fc gamma R-mediated phagocytosis	12	0.049062

### Network analysis

**Figure 3:**
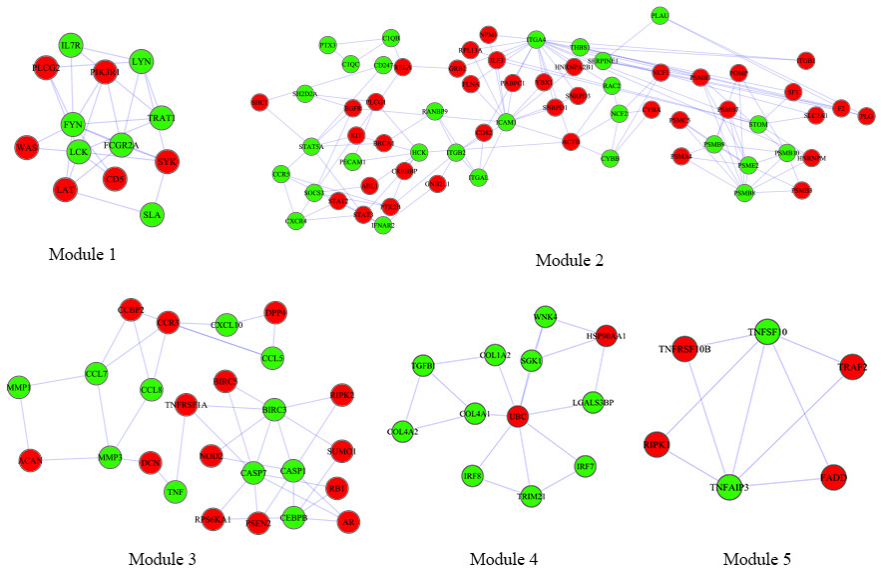
Modules of PPI network obtained through MCODE plugin of Cytoscape Red and green nodes represent DEGs and non differential expression genes respectively.

The 915 DEGs were found to be involved in 11466 PPI pairs in the union data of HPRD and BioGrid database. FBXO6 has the highest number, i.e. 614, of direct neighborhoods (which referred as degree hereinafter) in the PPI network and there are 14 genes, including BCL6, FOS, IL7R, CEBPB, LCK, ICAM1, STAT1, ISG15, FYN, LYN, UBC, VCAM1, ITGA4 and FBXO6, were found to have degree larger than 100. Besides, modular analysis resulted in 5 network modules with module score > 1.5. Table 3 lists the 5 modules and genes contained in them and Figure 3 illustrated the visualization of modules.

**Table 3:** The modules we obtained from the PPI network

Module ID	Score^a^	Nodes^b^	Edges^c^	Genes
1	2.154	13	28	CD5, FCGR2A, FYN, IL7R, LAT, LCK, LYN,PIK3R1, PLCG2, SLA, SYK, TRAT1, WA
2	1.894	66	125	ABL1, ACTB, BRCA1, C1QB, C1QC, CCR5, CD247, CD82, CREBBP, CXCR4, CYBA, CYBB, EGFR, F2, FLNA, GNB2L1, GRB2, HCK, HNRNPA2B1, HNRNPM, ICAM1, IFNAR2, ILF3, ITGA4, ITGAL, ITGB1, ITGB2, KIT, NCF1, NCF2, NPM1, PABPC1, PECAM1, PLAU, PLCG1, PLG, POMP, PSMA4, PSMB1, PSMB10, PSMB3, PSMB7, PSMB8, PSMB9, PSMC5, PSME2, PTK2B, PTX3, RAC2, RANBP9, RELA, RPL13A, SERPINE1, SF1, SH2D2A, SHC1, SLC2A1, SNRPD1, SNRPD3, SOCS3, STAT2, STAT3, STAT5A, STOM, THBS1, YBX1
3	1.561	25	39	ACAN, AR, BIRC3, BIRC5, CASP1, CASP7, CCBP2, CCL5, CCL7, CCL8, CCR3, CEBPB, CXCL10, DCN, DPP4, MMP1, MMP3, NOD2, PSEN2, RB1, RIPK2, RPS6KA1, SUMO1, TNF, TNFRSF1A
4	1.522	12	18	COL1A2, COL4A1, COL4A2, HSP90AA1, IRF7, IRF8, LGALS3BP, SGK1, TGFBI, TRIM21, UBC, WNK4
5	1.514	6	9	FADD, RIPK1, TNFAIP3, TNFRSF10B, TNFSF10, TRAF2

Note: a: The score of a module

b: The number of genes in the module

c: The number of interactions in the module

### qPCR and Western blot confirm genes that involved in the RAS pathological process

**Figure 4:**
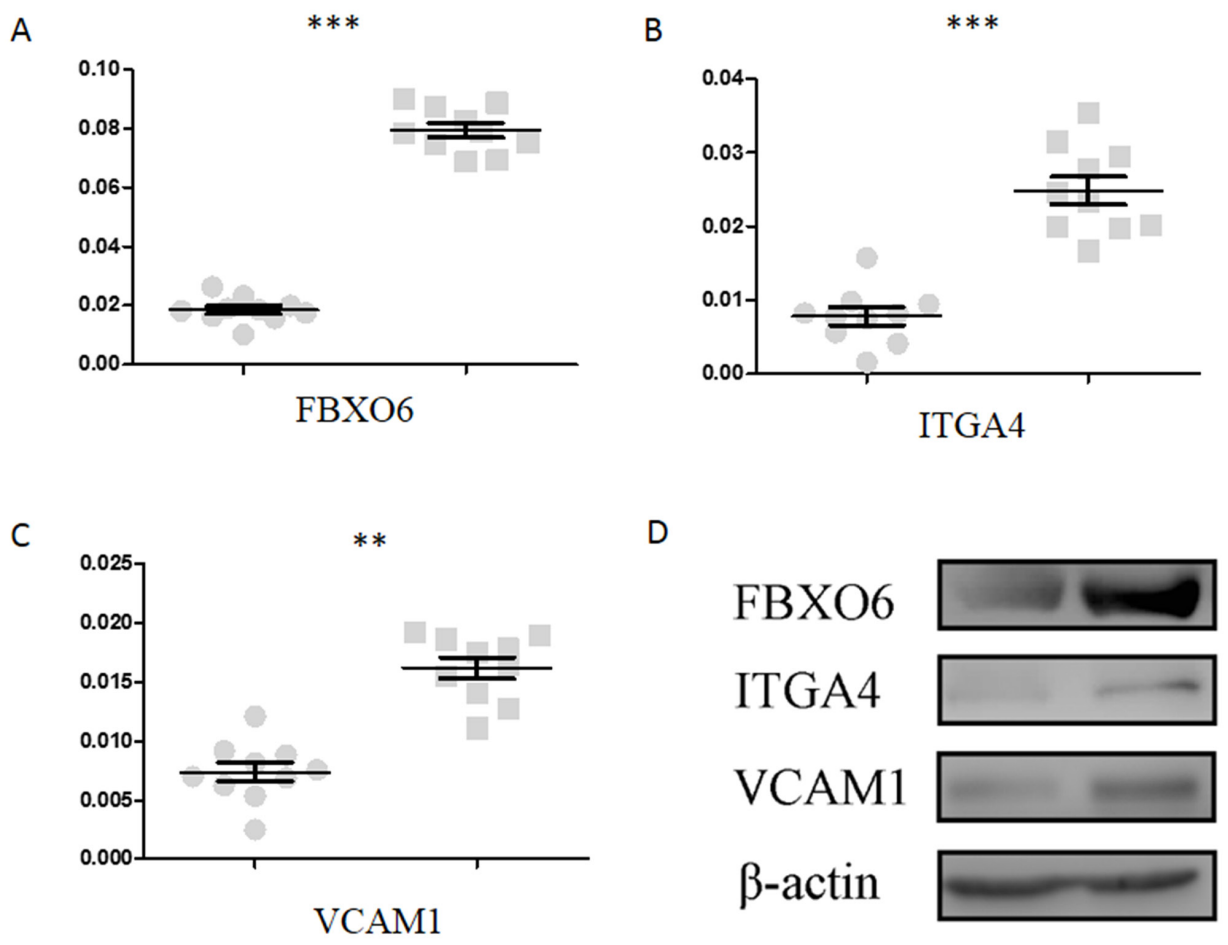
Validation of FBXO6, ITGA4 and VCAM1 through qPCR and Western blot **(A)**, **(B)** and **(C)** is the relative mRNA level of FBXO6, ITGA4 and VCAM1 quantified by qPCR respectively. **(D)** Protein abundance of FBXO6, ITGA4 and VCAM1 detected by Western blot.

To validate the critical genes participate in the pathogenesis of RAS, ulcerated tissues from RAS patients and normal tissues from healthy donors were subjected to qPCR (Figure 4A, 4B, 4C) and Western blot (Figure 4D). The increased expression levels of FBXO6, ITGA4 and VCAM were verified to be consistent with the microarray results.

## DISCUSSION

Recurrent aphthous stomatitis (RAS), also known as canker sores and recurrent aphthous ulceration, is a recurrent oral mucosal disease that shows different geographical incidence rates in the world [17]. RAS is known to be extremely aching, and it may even have a negative effect on the quality of life of the affected individual, impairing eating, swallowing and speaking [18]. High-throughput gene expression analysis not only identifies genes that have a potential effect on disease, but also provides an understanding of expression changes during disease progression. In present study, by the comparison of gene expression profile from 14 case samples and 19 control samples, we identified 915 DEGs with adjPval<0.05 and |logFC|>1 including 707 up-regulated genes and 208 down-regulated genes.

GO enrichment analysis revealed that DEGs significantly enriched in immune response, inflammatory response and defense response. The healing of an ulcer is a dynamic process usually involving the classic three phases of wound healing known as inflammation, proliferation and remodeling [19]. ITGA4 encodes a member of the integrin alpha chain family of proteins. Integrins are heterodimeric integral membrane proteins composed of an alpha chain and a beta chain that function in cell surface adhesion and signaling [20]. The encoded preproprotein is proteolytically processed to generate light and heavy chains that comprise the alpha 4 subunit. This subunit associates with a beta 1 or beta 7 subunit to form an integrin that may play a role in inflammatory disease [21]. This integrin is a therapeutic target for the treatment of inflammation, including multiple sclerosis, Crohn’s disease and inflammatory bowel disease. This gene is a member of the Ig superfamily and encodes a cell surface sialoglycoprotein expressed by cytokine-activated endothelium. This type I membrane protein mediates leukocyte-endothelial cell adhesion and signal transduction, and may play a role in the development of artherosclerosis and rheumatoid arthritis [22]. VCAM1 interacts with integrin alpha-4/beta-1 (ITGA4/ITGB1) on leukocytes, and mediates both adhesion and signal transduction. The VCAM1/ITGA4/ITGB1 interaction may play a pathophysiologic role both in immune responses and in leukocyte emigration to sites of inflammation [23]. What’s more, proliferation is also very important during the healing of an ulcer. FBXO6 encodes a member of the F-box protein family which is characterized by an approximately 40 amino acid motif, the F-box. The F-box proteins constitute one of the four subunits of the ubiquitin protein ligase complex called SCFs (SKP1-cullin-F-box), which function in phosphorylation-dependent ubiquitination [24]. The F-box proteins are divided into 3 classes: Fbws containing WD-40 domains, Fbls containing leucine-rich repeats, and Fbxs containing either different protein-protein interaction modules or no recognizable motifs. The protein encoded by this gene belongs to the Fbxs class, and its C-terminal region is highly similar to that of rat NFB42 (neural F Box 42 kDa) which may be involved in the control of the cell cycle [25]. Li and his colleagues report that FBXO6 can significantly promote the growth and proliferation of gastric cancer cells and normal gastric cells and change the cell cycle of them [26].

There were still many deficiencies in our research. For example, only a few genes were confirmed. In future researches, we will further validate the reliable biomarkers for RAS by more functional search. Our final attempts are to find the reliable biomarkers for clinical examination.

## MATERIALS AND METHODS

### Gene expression profiles

The RAS gene expression profiles were downloaded from the Gene Expression Omnibus (GEO) with deposited by Baccaglini et al with the accession number of GSE37265 (https://www.ncbi.nlm.nih.gov/geo/query/acc.cgi?acc=GSE37265). A total of 33 samples, i.e. 19 adjacent non-ulcer tissue and 14 RAS samples were included in the dataset. The commercial Affymetrix Human Genome U133 Plus 2.0 Array (GEO accession no.: GPL570) was used for the quantification of genome-wide gene expression values.

### Differential expression analysis

Gene microarray analysis was all conducted through R programming software. Briefly, raw CEL data were imported into R through affy version1.54.0 package [10] and performed Robust Multi-Array (RMA) based correction for the comparable of expression values among all samples; the normalized expression profiles were then used for the identification of differential expression genes (DEGs) in RAS samples compared with adjacent non-ulcer tissues through linear models for microarray data (limma) version3.32.2 [11]. Genes with fold change > 2 and FDR < 0.05 were considered as significant.

### Functional enrichment analysis

We performed functional enrichment analysis for DEGs through the Database for Annotation, Visualization and Integrated Discovery (DAVID) to uncover biological processes involved in RAS. Gene Ontology (GO) terms and Kyoto Encyclopedia of Genes and Genomes (KEGG) pathways with p-value < 0.05 and minimum hits > 10 were considered as significantly enriched [12].

### Network analysis

To explore interaction pairs among DEGs, we obtained their protein-protein interaction (PPI) pairs from the union data in HPRD and BioGRID database [13, 14]. Cytoscape software [15] was used for the visualization of PPI network. Besides, for the interpretation of whole network, we performed modular analysis for the network based on the MCODE plugin [16] of Cytoscape based on the thresholds of module score > 1.5.

### Quantitative PCR

Total RNA was extracted from ulcerated tissues and normal tissues using EasyPure RNA Kit (TransGen Biotech) following the manufacturer’s protocol, and then subjected to reverse transcription PCR by EasyScript Reverse Transcriptase kit (TransGen Biotech).

The 7500 fast RT-PCR (ABI) was used for real-time quantitative PCR (qPCR). Three assays were carried out for each reaction tube. Data were analyzed with the 2−ΔΔCt method using GAPDH as internal control. Primers: FBXO6-f: ATCCT ACGAA ATGTG CCTCA AG, FBXO6-r: CCAAC ACGAA GTAGT CAGCC G; ITGA4-f: CACAA CACGC TGTTC GGCTA, ITGA4-r: CGATC CTGCA TCTGT AAATC GC; VCAM1-f: GGGAA GATGG TCGTG ATCCT T, VCAM1-r: TCTGG GGTGG TCTCG ATTTT A.

### Western blot

20μg proteins were separated on a PAGE-gel and then transferred to nitrocellulose. The filters were blocked with 5% nonfat dry milk powder in TBS. Primary Abs were diluted 1/1000 in TBS containing 3 mg/ml BSA and 0.02% sodium azide, and incubated with the filters overnight at 4°C. After washing with TBST, the filters were incubated 1h with HRP-conjugated goat anti-rabbit IgG. Filters were washed extensively with TBST, and immunoreactive bands were visualized by ECL.

## References

[1] Izakovicova Holla L, Valova S, Borilova Linhartova P, Bartova J, Petanova J, Kuklinek P, Fassmann A (2017). Association study of interleukin-1 family, interleukin-6, and its receptor gene polymorphisms in patients with recurrent aphthous stomatitis. J Oral Pathol Med.

[2] Yilmaz HG, Albaba MR, Caygur A, Cengiz E, Boke-Karacaoglu F, Tumer H (2017). Treatment of recurrent aphthous stomatitis with Er,Cr:YSGG laser irradiation: A randomized controlled split mouth clinical study. J Photochem Photobiol B.

[3] Suter VG, Sjölund S, Bornstein MM (2017). Effect of laser on pain relief and wound healing of recurrent aphthous stomatitis: a systematic review. Lasers Med Sci.

[4] Chavan M, Jain H, Diwan N, Khedkar S, Shete A, Durkar S (2012). Recurrent aphthous stomatitis: a review. J Oral Pathol Med.

[5] Najafi S, Yousefi H, Mohammadzadeh M, Bidoki AZ, Firouze Moqadam I, Farhadi E, Amirzargar AA, Rezaei N (2015). Association study of interleukin-1 family and interleukin-6 gene single nucleotide polymorphisms in recurrent aphthous stomatitis. Int J Immunogenet.

[6] Buño IJ, Huff JC, Weston WL, Cook DT, Brice SL (1998). Elevated levels of interferon gamma, tumor necrosis factor alpha, interleukins 2, 4, and 5, but not interleukin 10, are present in recurrent aphthous stomatitis. Arch Dermatol.

[7] Wray D, Graykowski EA, Notkins AL (1981). Role of mucosal injury in initiating recurrent aphthous stomatitis. Br Med J (Clin Res Ed).

[8] Schulze A, Downward J (2001). Navigating gene expression using microarrays--a technology review. Nat Cell Biol.

[9] Borra RC, Andrade PM, Silva ID, Morgun A, Weckx LL, Smirnova AS, Franco M (2004). The Th1 /Th2 immune-type response of the recurrent aphthous ulceration analyzed by cDNA microarray. J Oral Pathol Med.

[10] Gautier L, Cope L, Bolstad BM, Irizarry RA (2004). affy--analysis of Affymetrix GeneChip data at the probe level. Bioinformatics.

[11] Diboun I, Wernisch L, Orengo CA, Koltzenburg M (2006). Microarray analysis after RNA amplification can detect pronounced differences in gene expression using limma. BMC Genomics.

[12] Huang W, Sherman BT, Lempicki RA (2009). Systematic and integrative analysis of large gene lists using DAVID bioinformatics resources. Nat Protoc.

[13] Keshava Prasad TS, Goel R, Kandasamy K, Keerthikumar S, Kumar S, Mathivanan S, Telikicherla D, Raju R, Shafreen B, Venugopal A, Balakrishnan L, Marimuthu A, Banerjee S (2009). Human Protein Reference Database--2009 update. Nucleic Acids Res.

[14] Chatr-Aryamontri A, Oughtred R, Boucher L, Rust J, Chang C, Kolas NK, O’Donnell L, Oster S, Theesfeld C, Sellam A, Stark C, Breitkreutz BJ, Dolinski K, Tyers M (2017). The BioGRID interaction database: 2017 update. Nucleic Acids Res.

[15] Shannon P, Markiel A, Ozier O, Baliga NS, Wang JT, Ramage D, Amin N, Schwikowski B, Ideker T (2003). Cytoscape: a software environment for integrated models of biomolecular interaction networks. Genome Res.

[16] Bader GD, Hogue CW (2003). An automated method for finding molecular complexes in large protein interaction networks. BMC Bioinformatics.

[17] Hamedi S, Sadeghpour O, Shamsardekani MR, Amin G, Hajighasemali D, Feyzabadi Z (2016). The Most Common Herbs to Cure the Most Common Oral Disease: Stomatitis Recurrent Aphthous Ulcer (RAU). Iran Red Crescent Med J.

[18] Vaillant L, Samimi M (2016). [Aphthous ulcers and oral ulcerations]. Presse Med.

[19] Pavlić V, vujić-Aleksić V, Aoki A, Nežić L (2015). Treatment of recurrent aphthous stomatitis by laser therapy: A systematic review of the literature. Vojnosanit Pregl.

[20] Shishido S, Bönig H, Kim YM (2014). Role of integrin alpha4 in drug resistance of leukemia. Front Oncol.

[21] Kritas SK, Saggini A, Cerulli G, Caraffa A, Antinolfi P, Pantalone A, Rosati M, Tei M, Speziali A, Saggini R, Frydas A, Conti P (2014). Impact of mast cells on multiple sclerosis: inhibitory effect of natalizumab. Int J Immunopathol Pharmacol.

[22] Schwab N, Schneider-Hohendorf T, Wiendl H (2015). Therapeutic uses of anti-α4-integrin (anti-VLA-4) antibodies in multiple sclerosis. Int Immunol.

[23] Scalici JM, Arapovic S, Saks EJ, Atkins KA, Petroni G, Duska LR, Slack-Davis JK (2017). Mesothelium expression of vascular cell adhesion molecule-1 (VCAM-1) is associated with an unfavorable prognosis in epithelial ovarian cancer (EOC). Cancer.

[24] Chen X, Duan LH, Luo PC, Hu G, Yu X, Liu J, Lu H, Liu B (2016). FBXO6-Mediated Ubiquitination and Degradation of Ero1L Inhibits Endoplasmic Reticulum Stress-Induced Apoptosis. Cell Physiol Biochem.

[25] Hwang GW, Du K, Takahashi T, Naganuma A (2011). Inhibition of F-box protein FBXO6 gene expression by RNA interference enhances cadmium toxicity in HEK293 cells. J Toxicol Sci.

[26] Zhang L, Hou Y, Wang M, Wu B, Li N (2009). A study on the functions of ubiquitin metabolic system related gene FBG2 in gastric cancer cell line. J Exp Clin Cancer Res.

